# 3D-printed aerogels as theranostic implants monitored by fluorescence bioimaging

**DOI:** 10.1016/j.bioactmat.2024.07.033

**Published:** 2024-08-08

**Authors:** Ana Iglesias-Mejuto, Rui Pinto, Pedro Faísca, José Catarino, João Rocha, Luisa Durães, Maria Manuela Gaspar, Catarina Pinto Reis, Carlos A. García-González

**Affiliations:** a AerogelsLab, I+D Farma Group (GI-1645), Department of Pharmacology, Pharmacy and Pharmaceutical Technology, Faculty of Pharmacy, iMATUS and Health Research Institute of Santiago de Compostela (IDIS), Universidade de Santiago de Compostela, E-15782, Santiago de Compostela, Spain; b Research Institute for Medicines (iMed. ULisboa), Faculty of Pharmacy, Universidade de Lisboa, Av. Professor Gama Pinto, 1649-003, Lisboa, Portugal; c Joaquim Chaves Saúde, Joaquim Chaves Laboratório de Análises Clínicas, Miraflores, 1495069, Algés, Portugal; d CECAV-Faculty of Veterinary Medicina- Lusófona University- Lisbon University Center, Campo Grande 376, 1749-024, Lisboa, Portugal; e Faculty of Veterinary Medicina- Lusófona University- Lisbon University Center, Campo Grande 376, 1749-024, Lisboa, Portugal; f University of Coimbra, CERES-Chemical Engineering and Renewable Resources for Sustainability, Department of Chemical Engineering, 3030-790, Coimbra, Portugal; g Instituto de Biofísica e Engenharia Biomédica (IBEB), Faculdade de Ciências, Universidade de Lisboa, Campo Grande, 1749-016, Lisboa, Portugal

**Keywords:** Upconversion nanoparticles, Aerogels, Theranostic implants, *In vivo* fluorescence

## Abstract

Aerogel scaffolds are nanostructured materials with beneficial properties for tissue engineering applications. The tracing of the state of the aerogels after their implantation is challenging due to their variable biodegradation rate and the lack of suitable strategies capable of *in vivo* monitoring the scaffolds. Upconversion nanoparticles (UCNPs) have emerged as advanced tools for *in vitro* bioimaging because of their fluorescence properties. In this work, highly fluorescent UCNPs were loaded into aerogels to obtain theranostic implants for tissue engineering and bioimaging applications. 3D-printed alginate-hydroxyapatite aerogels labeled with UCNPs were manufactured by 3D-printing and supercritical CO_2_ drying to generate personalize-to-patient aerogels. The physicochemical performance of the resulting structures was evaluated by printing fidelity measurements, nitrogen adsorption-desorption analysis, and different microscopies (confocal, transmission and scanning electron microscopies). Stability of the aerogels in terms of physicochemical properties was also tested after 3 years of storage. Biocompatibility was evaluated *in vitro* by different cell and hemocompatibility assays, *in ovo* and *in vivo* by safety and bioimaging studies using different murine models. Cytokines profile, tissue index and histological evaluations of the main organs unveiled an *in vivo* downregulation of the inflammation after implantation of the scaffolds. UCNPs-decorated aerogels were first-time manufactured and long-term traceable by fluorescence-based bioimaging until 3 weeks post-implantation, thereby endorsing their suitability as tissue engineering and theranostic nanodevices (i.e. bifunctional implants).

## Highlights

• Theranostic implants were manufactured by a dual processing strategy combining 3D-printing and supercritical CO_2_ drying.
• UCNPs-decorated aerogels were physicochemically stable and highly fluorescent after 3 years of storage.
• *In vitro* cell studies and hemocompatibility assays demonstrated the suitability of the 3D-printed aerogels for tissue engineering.
• *In vivo* tests showed the biocompatibility and the monitoring capability of the theranostic implants.

## Graphical abstract

**Figure:**
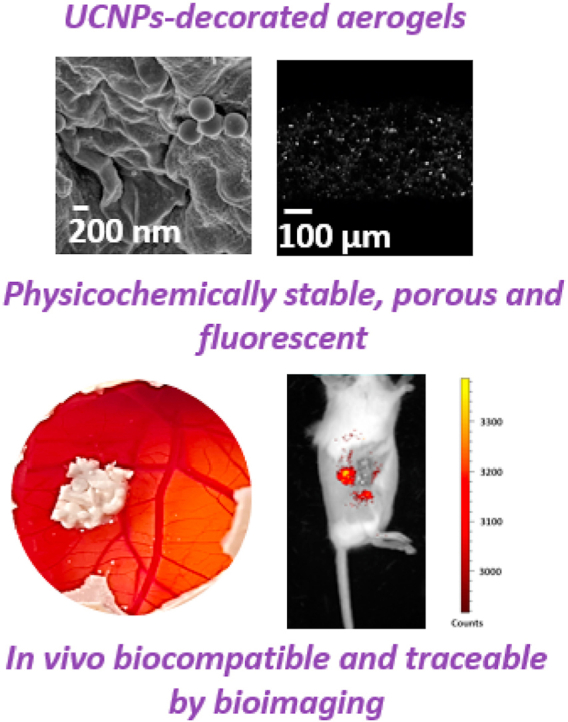

## 1 Introduction

Bioimaging allows the cellular and molecular study of disorders by the *in vivo* detection, visualization, quantification and tracing of biological and pathological changes targeted by a fluorescent probe [1,2]. Different non-invasive techniques such as positron emission tomography, magnetic resonance, computed tomography, fluorescence, Raman, optical or ultrasound imaging as well as photodynamic, photothermal, and photoacoustic methods are employed as tools for molecular imaging [3,4]. Namely, in fluorescence-based bioimaging, fluorophores are activated at specific excitation wavelengths in a safe and non-invasive way [5,6]. Visible light has a tissue penetration of only few millimeters, so near-infrared (NIR) fluorescent probes are under evaluation to increase the penetration capability. NIR light is non-toxic and induces low optical damage and self-fluorescence while allowing a rapid data collection and a precise control over the irradiated region. Accordingly, NIR-responsive probes are able to reach the region of interest to monitor the progress of pathologies and have been incorporated into nanodevices to significantly enhance regeneration of certain tissues like bone [5,7,8].

Contrast agents such as small molecular dyes, quantum dots, carbon nanotubes or nanodots are used for NIR bioimaging of tumor cells, vasculature, or vital organs thus enabling their anatomy and function evaluation by an image-guided therapy [6,9]. An ideal fluorescent probe may be biocompatible and stable while presenting a high signal-to-noise ratio and a low disruption effect on cell function [3]. Nanoparticles (NPs) from silica, metals or polymers conjugated with contrast agents have been studied as bioimaging tools [4]. NPs can effectively transport imaging probes to target tissues for monitoring and treating their state thereby presenting diagnostic and therapeutic capabilities simultaneously [3]. For these reasons, nanodevices usually replace traditional molecular probes in bioimaging applications reaching preclinical and clinical trials [4]. Namely, the advanced photophysical properties of upconversion NPs (UCNPs) make them excellent candidates for non-invasive intravital imaging and diagnosis thereby translating their *in vitro* NIR-responsive fluorescence into an *in vivo* imaging approach [10]. Lanthanides are commonly used to synthesize UCNPs due to their luminescent properties, such as high stability, long lifetimes and narrow emission bands [11]. UCNPs composition typically also includes LaF_3_, BaYF_5_ or NaYF_4_ as host matrices, Yb^3+^ or Nd^3+^ as dopants, and Er^3+^ or Ho^3+^ as NIR-absorption enhancers [12]. Highly fluorescent and photostable UCNPs could be integrated into scaffolds to finally obtain 3D-nanostructures with diagnostic and therapeutic properties. The combination of UCNPs and tissue engineering scaffolds could thereby result in a bifunctional implant that allows simultaneously the dynamic monitoring and the effective promotion of tissue regeneration.

Biomaterials sought for tissue engineering applications must be able to mimic the extracellular matrix while promoting cell attachment, proliferation and migration [[13], [14], [15]]. High porosity, surface area and loading capacity are thus desired properties for biomaterials designed as advanced carriers for imaging probes [4]. Aerogels are nanostructured materials that can be manufactured adapted-to-patient with a dual porosity and high entrapment yield of drugs, nanofibers and NPs by a dual processing strategy combining 3D-printing and supercritical carbon dioxide (scCO_2_) technologies [16,17]. These highly porous structures are promising tissue engineering platforms as they present personalized external shape, internal pattern and composition, while being biocompatible, bioactive, and promoters of long-term cell adhesion and migration [18,19]. The tracing of the degradation profile of biomaterials once implanted has been studied by non-invasive imaging, but is challenging when the growth of new tissue is simultaneously taking place [8]. For this purpose, fluorescent probes can enhance the accuracy of the monitoring process while being easy to synthesize, non-toxic and labeled to biodegradable materials [5,20]. Fluorescent aerogels have been proposed as novel optical sensing probes [21,22] but, to the best of our knowledge, advanced NIR-responsive systems like UCNPs doped aerogels have not been designed and tested yet. Highly fluorescent aerogel materials suitable for bioimaging applications have been thus theoretically proposed [21,22], but not practically explored as advanced theranostic biomaterials.

In this work, NIR-responsive 3D-biomaterials were manufactured from dual porous aerogels and highly fluorescent UCNPs for the first time. The effective incorporation of UCNPs into 3D-printed alginate aerogels resulted in advanced highly porous scaffolds with potential to regenerate the damaged tissue while monitoring the state and position of the implanted device. Physicochemical characterization of UCNPs-doped aerogels was studied by nitrogen adsorption-desorption analysis, and microscopy evaluations (scanning electron -SEM-, transmission electron -TEM- and confocal microscopies). Biological performance of UCNPs-labeled aerogels was assessed by biocompatibility, migration, and adhesion cell studies. Hemocompatibility of the implants was evaluated by hemolytic activity measurements and Hen's egg test on the chorioallantoic membrane (HET-CAM). *In vivo* safety assessment of the theranostic aerogel-based materials was performed by evaluating the expression of tumor necrosis alpha factor (TNF-α) and interleukins 6 (IL-6) and 10 (IL-10) as well as the histology of the main organs 1 week after subcutaneous implantation in two murine models. Finally, a 3-week bioimaging *in vivo* study was carried out in mice to unveil the monitoring capability of the UCNPs loaded into these aerogel-based biomaterials.

## 2 Materials and methods

### 2.1 Materials

Alginic acid sodium salt from brown algae with medium viscosity (guluronic acid/mannuronic acid ratio: 70/30, Mw 403 kDa, 3170 cps), calcium chloride (CaCl_2_; Mw 110.98 g/mol, 99.99 % purity), ytterbium(III) nitrate pentahydrate (Yb(NO_3_)_3_·5H_2_O, 99.9 % purity), yttrium(III) nitrate hexahydrate (Y(NO_3_)_3_·6H_2_O, 99.8 % purity), ethylenediaminetetraacetic acid (EDTA) and sodium fluoride (99 %) were obtained from Sigma Aldrich (Madrid, Spain). Erbium(III) nitrate pentahydrate (Er(NO_3_)_3_·5H_2_O, 99.9 % purity), and tetraethyl orthosilicate (TEOS, Si(OC_2_H_5_)_4_, 98 % purity), were supplied by Acros Organics (Thermo-Fisher Scientific, Geel, Belgium). Hydroxyapatite (HA; Mw 502.31 g/mol, micropowder) was obtained from Fluidinova (Moreira da Maia, Portugal). Ammonium hydroxide (25 % NH_3_ in H_2_O) and hydrogen peroxide (H_2_O_2_; 30 % aqueous solution) were supplied by Honeywell Fluka (Madrid, Spain). CO_2_ (purity >99.9 %) was provided by Nippon Gases (Madrid, Spain) and absolute ethanol (EtOH) by VWR (Radnor, PA, USA). Water was purified by reverse osmosis (resistivity >18 MΩ·cm; Milli-Q, Millipore®, Madrid, Spain) before further use.

### 2.2 Synthesis of highly luminescent UCNPs by co-precipitation and sol-gel methods

Core-shell UCNPs with a silica shell were synthesized following a procedure described on a previous work from our team [10]. Briefly, 16 mL of 0.2 M Y(NO_3_)_3_, 3.4 mL of 0.2 M Yb(NO_3_)_3_, 0.6 mL of 0.2 M Er(NO_3_)_3_ and 20 mL of 0.2 M EDTA were mixed and injected into 60 mL of 0.8 M NaF aqueous solution. The obtained solution was stirred 1 h at room temperature (RT) and the resulting dispersions were centrifuged and washed three times with water and once with ethanol. Afterwards, NPs were dried in an oven at 60 °C. Annealing was then performed for 5 h, under a N_2_ atmosphere, at 400 °C (heating rate: 20 °C/min). NPs were cooled down until RT under the same atmosphere. To obtain the silica shell, 30 mL of NPs dispersed in ethanol (3 mg/mL) were stirred at 0 °C with 0.2 mL of TEOS and, after 5 min, 3 mL of ammonium hydroxide was added. Reaction took place for 2 h and the thus obtained UCNPs were washed three times with EtOH.

### 2.3 3D-printing of UCNPs-labeled alginate gels

6 wt% alginate solution was prepared at RT and 600 rpm, using a homogenizer (VWR vos 60, Radnor, PA, USA) and 0.4 wt% or, alternatively, 0.8 wt% of UCNPs were added to the Alg-based ink to compare their fluorescence and physicochemical behavior. For the comparison of textural properties, formulations with increasing content of HA were obtained by adding 8, 16 or 24 wt% of HA to the 6 wt% alginate solution with or without 0.4 wt% of UCNPs. The formulation with 24 wt% of HA was simply denoted as AlgHA, without specifying the wt.% of HA. The same ink formulations without UCNPs or HA was used as blank [19]. All inks were degassed for 10 min to eliminate air bubbles in a sonication bath (Branson 3510 Emerson, Ferguson, MO, USA). The filament drop test was performed and the general pore shape geometry was also evaluated to assess the printability of the different inks through a 410-μm nozzle [23].

Hydrogels were manufactured by 3D-printing of inks at RT with a Cellink BIO X Bioprinter (Boston, MA, USA), employing an extrusion printhead with a 3-mL syringe and a 410-μm nozzle. A printing pressure of 60 kPa, printing velocity of 12 mm/s and a grid pattern of 2 × 2 × 0.1 cm^3^ with three layers, were the set parameters to print all formulations tested. After 3D-printing, hydrogels were put in contact with 1 M CaCl_2_ aqueous bath for 1 h and then with EtOH for solvent exchange.

### 2.4 Rheological evaluation of inks

Flow behavior of the inks was studied at RT from 0.05 to 200 rad/s in a Rheolyst AR-1000N rheometer (TA Instruments, Newcastle, UK) with a Peltier plate and a cone geometry (40 mm diameter, 2°). At least four replicates per formulation were evaluated. The power law (Equation (1)) was fitted to the linear region of the viscosity (η) vs shear rate (γ) plots to assess the shear thinning behavior of the inks [24].

(1) η = bγ^c^

where *b* and *c* are the consistency and flow coefficients, respectively.

### 2.5 scCO_2_ drying of 3D-printed gels

Alcogels were wrapped in filter paper, placed into a 100-mL stainless steel autoclave (Thar Process, Pittsburg, PA, USA) and immersed in 20 mL of EtOH. CO_2_ was supplied with a dual-piston pump from the top of the vessel at 40 °C. A CO_2_ flow rate (5–7 g/min) at 120 bar passed through the autoclave for 4 h. After a CO_2_ depressurization at 2 bar/min, aerogels were collected from the autoclave for further use. Aerogels were denoted as “Alg (HA) UCNPs *x”* being *x* the content of UCNPs in the ink (0.4 or 0.8 wt%). Aerogels prepared only with Alg (i.e. without UCNPs or HA) were obtained as controls and simply denoted as “Alg”. The composition of the formulations studied are described in Table 1.

**Table 1:** Aerogel formulations with or without UCNPs studied.

Formulation	Ink composition		
	Alg (wt. %)	HA (wt. %)	UCNPs (wt. %)
Alg	6	–	–
AlgHA8	6	8	–
AlgHA16	6	16	–
AlgHA	6	24	–
Alg UCNPs 0.4	6	–	0.4
Alg UCNPs 0.8	6	–	0.8
AlgHA8 UCNPs 0.4	6	8	0.4
AlgHA16 UCNPs 0.4	6	16	0.4
AlgHA UCNPs 0.4	6	24	0.4

### 2.6 scCO_2_ sterilization of 3D-printed gels

3D-printed UCNPs-decorated aerogels were introduced into sterilization pouches and were placed in a 600-mL autoclave (NovaGenesis, NovaSterilis Inc., Ithaca, NY, USA) with 1200 ppm of H_2_O_2_. The system was heated until 40 °C, pressurized with CO_2_ until 100 bar, these conditions maintained for 30 min and then depressurized at 5 bar/min. The physicochemical performance of the thus sterilized UCNPs-decorated aerogels was evaluated by BET and confocal microscopy in the 405–561 nm range (Leica TCS-SP2, Mannheim, Germany).

### 2.7 Physicochemical characterization of UCNPs-labeled aerogels

External dimensions of the gels before and after the scCO_2_ drying step were measured with a digital caliper (Fowler™, Newton, MA, USA) to assess the volume shrinkage by Equation (2) and the shape fidelity factor (SFF) by Equation (3):

(2) Volume shrinkage (%) = [(Alcogel volume − Aerogel volume)/Alcogel volume] × 100

(3) SFF = alcogel or aerogel printed area/ CAD area (printing file)

Low-temperature nitrogen adsorption-desorption analysis was used to evaluate the physicochemical performance of aerogels (ASAP 2000 Micromeritics Inc., Norcross, GA, USA). Briefly, aerogels were degassed under vacuum at 40 °C for 24 h and the specific surface area (A_BET_) was obtained by applying the BET (Brunauer-Emmett-Teller) method, while specific pore volume (V_p_), and mean pore diameter (d_p_) were obtained from the use of the BJH (Barrett–Joyner–Halenda) method. Aerogels were iridium-sputtered and then imaged by SEM and the chemical elements in the implants were mapped by SEM-EDX (EVO LS15, Zeiss, Oberkochen, Germany). UCNPs were imaged by TEM (TEM, JEOL JEM-2010, JEOL, Tokyo, Japan -operating at 200 kV-) and these images as well as the SEM images of the UCNPs loaded in aerogels were employed for particle size distribution analysis considering the diameter of at least 60 UCNPs. Attenuated Total Reflectance/Fourier-Transform Infrared Spectroscopy (ATR/FT-IR) was also performed in the mid-IR spectrum range (400–4000 cm^−1^) to assess the chemical structure of the aerogels. Fluorescence of UCNPs-labeled aerogels was studied by confocal microscopy evaluation.

In certain cases, aerogel scaffolds were placed in closed plastic jars at RT and protected from light for 3 years. After storage, the fluorescence and physicochemical long-term stability of UCNPs-doped aerogels were evaluated by nitrogen adsorption-desorption analysis, TEM, SEM and confocal microscopies.

### 2.8 Cellular studies

#### 2.8.1 Cell viability tests

Aerogels of 1 × 1 × 0.1 cm^3^ dimensions with and without UCNPs were UV-sterilized for 30 min before all cellular tests. Cytocompatibility of the UCNPs-labeled aerogels was evaluated by the WST-1 test, based on the degradation of WST-1 into formazan, which is correlated with the number of metabolically active cells. Mouse embryo fibroblasts (BALB/c 3T3, 6500 cells/cm^2^) were seeded in 24-well plates with 1500 μL of Dulbecco's Modified Eagle's Medium (DMEM) supplemented with 15 % fetal bovine serum, penicillin 100 U/mL and streptomycin 100 μg/mL. Cells were incubated at 37 °C in a humidified atmosphere enriched with 5 % CO_2_ and aerogels were placed in the bottom of culture inserts. Positive controls (cells with 1000 μL of DMEM) were maintained at the same conditions and all tests were run in triplicate. Aerogels were removed after 24 and 48 h of culture. Afterwards, 250 μL of the medium was left in the wells and 25 μL of WST-1 was added. The plate was incubated at the same conditions for 2 h. After shaking the plate for 1 min, 110 μL were poured into a 96-well and absorbance was measured at λ = 450 nm in a microplate reader (Infinite® M200, Tecan Group Ltd., Männedorf, Switzerland).

#### 2.8.2 Cell attachment tests

The spreading of human bone marrow mesenchymal stem cells (MSCs) on the aerogels with and without UCNPs was assessed by DAPI staining to detect the nuclei in triplicate. After 13 days of culture, aerogels were fixed with paraformaldehyde 4 % w/v and washed with PBS. After Triton/PBS (0.2 % v/v) incubation for 5 min, scaffolds were washed with PBS and placed on glass slides. One drop of the DAPI-containing ProLong gold antifade mountant (Molecular Probes Inc., Eugene, OR, USA) was poured on each scaffold before their storage at −20 °C. Cell attachment was evaluated with a confocal microscope (Leica TCS-SP2, Mannheim, Germany) and ImageJ software was used to analyze the images.

#### 2.8.3 Migration assay

Cell migration on the aerogels with and without UCNPs was assessed by a wound healing test. After confluence, BALB/c 3T3 cells monolayer was scratched by a straight line with a pipette tip to mimic a wound *in vitro*. Cells were then washed with PBS. Aerogels with and without UCNPs were placed in culture inserts in contact with cells in triplicate. Images of the wound were taken with an inverted microscope (Olympus, Tokyo, Japan) immediately and after 24 h to evaluate the cell migration. The migration area (%) was calculated with Eq. (4):

(4) Migration area (%) = (A_0_-A_n_) / A_0_ x 100

where A_0_ is the initial wound area and A_n_ is the wound area after 24 h.

### 2.9 Hemocompatibility studies

#### 2.9.1 Hemolytic activity test

Hemolytic activity of the UCNPs-labeled aerogels was tested with human blood (Galician Transfusion Center, Santiago de Compostela, Spain) obtained in accordance with the Declaration of Helsinki. Total blood was diluted to 33 % v/v in 0.9 % w/v NaCl solution and 1 mL was transferred to Eppendorf tubes containing either 1 × 1 × 0.1 cm^3^ aerogels, 100 μL of 4 % v/v Triton X-100 (positive control) or 100 μL of 0.9 % w/v NaCl (negative control). Tests were carried out in triplicate. Samples were incubated for 60 min at 37 °C and 100 rpm and then centrifuged for 10 min at 10,000*g* (Sigma 2-16P, Osterode am Harz, Germany). 150 μL of the supernatant were transferred to a 96-well plate, and the absorbance of the hemoglobin was measured at λ = 540 nm (FLUOStar Optima, BMG Labtech, Ortenberg, Germany). Hemolysis was calculated with Equation (5):

(5) Hemolysis (%) = (Abs_s_ − Abs_n_) / (Abs_p_ − Abs_n_) × 100

where Abs_s_ is the absorbance of the samples in contact with aerogels, Abs_n_ is the absorbance of the negative control, and Abs_p_ is the absorbance of the positive control.

#### 2.9.2 HET-CAM assay

HET-CAM test was performed according to a standard protocol [25] previously used to evaluate the hemocompatibility of implants [17]. The assay was performed with fresh, clean, and fertilized Hen's eggs (50–60 g, Coren, Ourense, Spain). Horizontal incubation took place in a climatic chamber (Ineltec CC SR 0150, Barcelona, Spain) at 37 °C, 60 % humidity and with a scheduled rotation after 8 h. On day 9, a small window of 1 cm diameter was opened in the eggshell to access the CAM. 300 μL of 0.1 N NaOH and of 0.9 % w/v NaCl were pipetted on the CAM as positive and negative controls, respectively. Aerogels with and without UCNPs were put in contact with the CAM and each sample was tested in triplicate. Vessels of the CAM were visually inspected for 5 min to assess the appearance of hemorrhage, vascular lysis, coagulation, hyperemia, or other changes in CAM vessels.

### 2.10 Animals care and subcutaneous implantation of scaffolds

#### 2.10.1 *In vivo* safety studies

Biocompatibility, degradation and functionality of biomaterials are usually firstly evaluated by surgical ectopic subcutaneous implantation in small animal models [26]. Mice model was chosen to evaluate the toxicity and the functionality of UCNPs-labeled aerogels as theranostic implants. Rat model is more relevant than mouse due to their articular cartilage structure that mimics better the one observed in humans, so it was selected to test formulations aimed at bone tissue engineering [26]. Wistar rats (male, 200–300 g) from Charles River (Barcelona) and mice BALB/c (male, 20–30 g) from IGC (Lisbon) were housed and maintained in compliance with the guidelines outlined in the Guide for the Care and Use of Laboratory Animals, in accordance with the national (DL 113/2013, 2880/2015, 260/2016 and 1/2019) and international (Directive 2010/63/EU) accepted principles for laboratory animals' use (3 R's principles). Safety studies were reviewed and approved by the DGAV (national authority) and by Animal Experiment Ethics Committee (ORBEA) of the Faculty of Pharmacy of University of Lisboa following the Declaration of Helsinki. All animals had free access to acidified water and were fed with a commercial rodent chow while being maintained at 22 ± 1 °C with a controlled 12 h light/dark cycle.

The study design consisted of 3 experimental groups: (1) 2 healthy mice (n = 2) with an implanted scaffold without UCNPs (Alg), (2) 2 healthy mice (n = 2) with an implanted scaffold with UCNPs (Alg UCNPs 0.8), and (3) 2 healthy mice (n = 2) employed as controls (surgery without scaffold implantation). Next, other 2 experimental groups were designed: (1) 2 healthy rats (n = 2) with a scaffold with HA and UCNPs (AlgHA UCNPs 0.4), and (2) 2 healthy rats (n = 2) employed as controls (surgery without scaffold implantation). Before implantation, the hair of the dorsal area where the implant was going to be placed was removed by using an electric shaver in all cases. Skin was prepared with 10 % povidone iodine and 70 % isopropanol. An incision of 1 cm was done, and a 1 × 1 × 0.1 cm^3^ scaffold was implanted on a subcutaneous pocket per animal. Incision was closed in a simple interrupted suture using silk (3-0) and (2-0) for rats and mice, respectively. The implantation of biomaterials in the dorsal skinfold chamber is commonly employed to assess the interaction between the biomaterials and their host tissue in rats and mice [27].

1-week post-surgery, animals were euthanized with isoflurane and implanted scaffolds were carefully removed and photographed to visually evaluate the connective-tissue capsule around and penetrating the aerogel scaffolds. Implants employed for all *in vivo* studies were sterilized by using a scCO_2_ method previously developed in our lab without causing a detrimental effect on the physicochemical performance of the 3D-printed aerogels [19] and fulfilling the requirements for the sterilization of implantable medical devices [28].

##### 2.10.1.1 Circulating cytokine quantification

Peripheral blood was obtained by cardiac puncture 1 week after implantation under isoflurane anesthesia. Blood was centrifuged and plasma was separated from the cellular fraction. The TNF-α, IL-6, and IL-10 expression levels were determined spectrophotometrically at 450 nm by employing an ELISA kit (Quantikine® Hycult Biotechnology, Wayne, PA, USA) and following manufacturer's instructions.

##### 2.10.1.2 Histological evaluation of major organs

The tissues of major organs (liver, spleen, and right kidney) were excised, and the tissue index was calculated by weighing the organs and using Equation (6).

(6) $$\text{Tissue}\;\text{Index}=\sqrt{\text{Organ}\;\text{Weight}/\text{Body}\;\text{Weight}}\times 100$$

The same tissues were subjected to histological evaluation to study the biocompatibility of implants [8]. The excised organs were fixed in 10 % v/v formalin, embedded in paraffin, and sectioned at 3 μm of thickness for hematoxylin-eosin (H&E) staining following a standard protocol to assess the morphological features of each tissue. Sections were examined under a microscope (Olympus BX51, Olympus Corporation, Tokyo, Japan) and images were acquired with a NanoZoomer-SQ Digital slide scanner (Hamamatsu Photonics, Shizouka, Japan). The histological evaluation was carried out by a veterinary pathologist in a blinded manner.

#### 2.10.2 *In vivo* bioimaging studies

Swiss mice (male, 20–30 g) were supplied by the animal facilities at the University of Santiago de Compostela (CEBEGA, Santiago de Compostela, Spain). All animals had free access to tap water and to a commercial rodent chow and were housed under controlled conditions of temperature (22 ± 1 °C), humidity (60 ± 5 %), and day-night cycles (12 h/12 h). Bioimaging studies were approved by the University of Santiago de Compostela Ethics Committee for Animal Experiments (Project Approval Number: 15012/2023/010) and followed the Spanish and European Union (EU) rules (86/609/CEE, 2003/65/CE, 2010/63/EU, RD 1201/2005, and RD53/2013) as well as the Declaration of Helsinki. The study design consisted of 3 healthy mice (n = 3) with 2 implanted scaffolds (one without -Alg- and other with UCNPs -Alg UCNPs 0.8-) and 2 healthy mice (n = 2) employed as control of fluorescence emission for all bioimaging experiments (surgery performed without scaffold implantation). Dorsal hair was clipped, and skin was prepared with iodine solution and 70 % isopropanol. Two longitudinal skin incisions of 1 cm were made per animal, located approximately 3 cm from the mid dorsal line and separated between them by 6 cm. One scaffold of 1 × 1 × 0.1 cm^3^ was placed on a lateral subcutaneous pocket at each incision. A single stainless-steel staple was used per incision to close each wound. Fluorescence images of animals were taken under deep-inhalation anesthesia with isoflurane at different timepoints (just after implantation, and after 4 h, 1 day and 1, 2 and 3 weeks) by using an In Vivo Imaging System (IVIS®, Caliper Life Sciences, Alameda, CA, USA). All images were acquired with an excitation wavelength of 745 nm and emission wavelength of 840 nm. The exposure time was 60 s per image and the same settings were used for all measurements. ImageJ software was used to measure the different regions of interest where fluorescence was detected at each timepoint, give a numerical value to each region and comparatively analyze and discuss the fluorescence profile obtained over time. 3 weeks after subcutaneous implantation of scaffolds, animals were sacrificed by breaking their cervical vertebra. The surrounding tissue where the scaffold was placed was opened with scissors and this skin area and the major organs were evaluated *ex vivo* regarding their fluorescence emission.

### 2.11 Statistical analysis

Results obtained from at least 3 experiments were denoted as mean value ± standard deviation. Post hoc Tukey HSD multiple comparison tests were performed to assess the statistical significance of the differences between groups and controls. Values of p < 0.05 were considered as statistically significant.

## 3 Results and discussion

### 3.1 Rheological assessment of inks

**Fig. 1:**
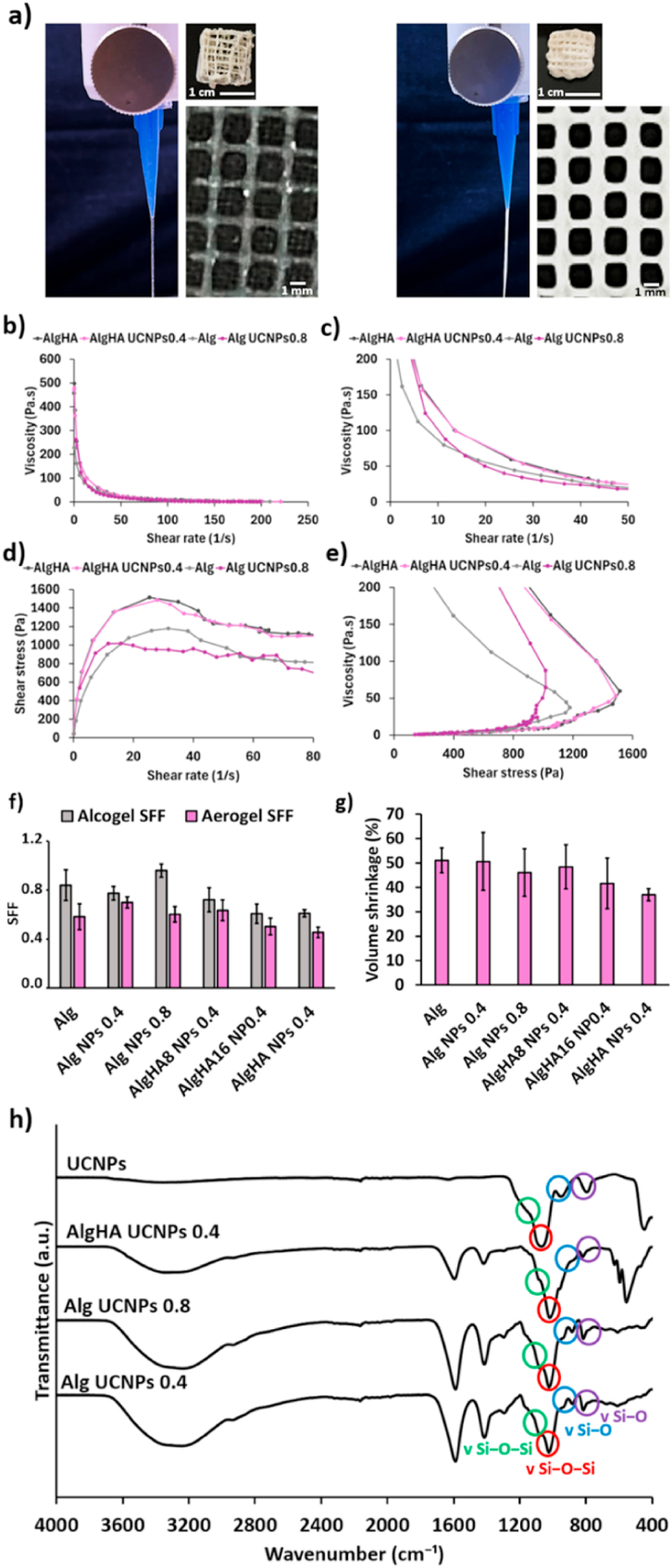
Physicochemical evaluation of UCNPs-decorated aerogels. **(a)** Filament drop test, general pore shape geometry and visual appearance of aerogels obtained from different ink formulations: Alg UCNPs 0.8 (left) and AlgHA UCNPs 0.4 (right). Rheological evaluation of inks by **(b,c)** overall viscosity with respect to the shear rate; **(d)** shear stress with respect to the shear rate, and **(e)** overall viscosity with respect to the shear stress. **(f)** SFF and **(g)** volume shrinkage (in percentage) of different UCNPs-decorated aerogels. No statistically significant differences among groups were obtained after post hoc Tukey HSD multiple comparison test (*p* < 0.05). **(h)** ATR/FT-IR spectra of UCNPs and aerogel formulations.

Ink printability requires a correct fiber formation and shear thinning properties [29]. The printability must be adapted to the 3D-printing process to manufacture gels with high shape fidelity. The filament drop test and the evaluation of the pore shape geometry have been reported as useful methods to predict ink printability and shape fidelity [23,29]. The different inks studied by the filament drop test formed continuous and straight fibers with good integrity of the 3D-printed filament just after extrusion (Fig. 1a). Squared and closed pores formed a coherent grid pattern without relevant ink spreading or filament fusion after deposition. All these findings have been previously linked to good printability [23,29,30]. Also, a uniform layer by layer structure was observed for the 3D-printed aerogels after all manufacturing process, regardless of the HA or UCNPs content.

All formulations presented similar shear thinning profiles, with a typical decrease in viscosity over a shear rate ramp (Fig. 1b and c), predicting a good printability [24]. This demonstrates that the incorporation of HA or UCNPs at the concentrations studied (up to 0.8 wt% for UCNPs and up to 24 wt% for HA) was possible without disrupting the printability of the inks during the 3D-printing process. A plot of shear stress with respect to shear rate showed a non-linear flow curve (Fig. 1d) characteristic of non-Newtonian fluids [31], while the viscosity over a shear stress ramp (Fig. 1e) exhibited the “at-rest” behavior of the inks [24]. Regarding the shear thinning coefficients, similar values were obtained for the different inks (Table 2), thereby suggesting a close printability for all of them and further endorsing that the printability of the inks is mainly affected by their alginate component. It should be noted that lower values of *b* coefficient were found when HA was not incorporated into inks, thus suggesting a higher extrudability of these formulations and probably due to their lower viscosity. Moreover, *c* values closer to zero were obtained for inks without UCNPs and HA incorporated at the same time, thus indicating a more pronounced shear thinning behavior and, consistently, a better suitability for extrusion-based 3D-printing. Similar *b* and *c* coefficient values were previously reported in an study of the flow behavior of different alginate inks containing HA also for scaffold fabrication [32].

**Table 2:** Shear thinning coefficients values obtained through power law model.

Aerogel formulations	Shear thinning coefficients	
	b (Pa·s)	c
Alg	654.5	4.4 x 10^−8^
Alg UCNPs 0.8	552.8	3.9 x 10^−8^
AlgHA	831.0	3.8 x 10^−8^
AlgHA UCNPs 0.4	851.6	5.3 x 10^−7^

### 3.2 Physicochemical evaluation of UCNPs-decorated aerogels

All aerogel formulations (Table 1) were obtained with a visually homogeneous dispersion of their different components (Alg, HA or UCNPs). No relevant differences in terms of the printability window for the formulations with or without UCNPs were observed, probably due to the low concentrations employed (0.4 and 0.8 wt%). 3D-printing process was performed without relevant clogging at concentrations of HA as high as 24 wt% in combination with alginate and UCNPs. The addition of HA has induced long-term bioactivity on the 3D-printed scaffolds while maintaining their geometry and integrity, which is of interest in scaffolds intended for bone tissue engineering [18,19]. High fidelity of the 3D-printed aerogels was observed with respect to the original CAD design, so good SFF values were obtained for all the formulations without statistically significant differences between the different groups studied (Fig. 1f). This result suggests that neither the HA nor the UCNPs incorporation into the implants affects the printing fidelity of the resulting aerogels. Alcogels exhibited better values of SFF than aerogels with the same composition, linked to the volume shrinkage during the drying step undergone for all the formulations (Fig. 1g). All UCNPs-labeled aerogels showed values of volume shrinkage close to the same formulations without UCNPs, so volumetric shrinkage is not related to the UCNPs content. Nevertheless, a decreasing tendency in the volume shrinkage was observed when HA was incorporated into UCNPs-loaded aerogels, thereby suggesting that the HA promotes a lower shrinkage of the aerogels. Same tendency was previously described for aerogels with the same composition but without UCNPs [18,19], thus endorsing the neglectable effect of the UCNPs in the morphological performance of aerogels.

The FT-IR spectra of the Alg aerogels showed the characteristic peaks of alginate in the 1400-4000 cm^−1^ region for all aerogel formulations studied (Fig. 1h). Namely, the band at 1646 cm^−1^ results from the interaction of the carboxylic groups of the alginate with the Ca^2+^ ions of the crosslinker used. The band at 1587 cm^−1^ denotes the asymmetric stretching vibration (-C=O) due to the carboxylic acid salts related to the ionic crosslinking [33,34]. The broad band around 3252 cm^−1^ (O–H stretching vibration) and the one at 1407 cm^−1^ (symmetric COO stretching vibration), are also related to alginate [35]. In the UCNPs spectrum, bands related to the silica coating appeared in the 800-1080 cm^−1^ region [10]. The broad peaks at 1080 and at 1175 cm^−1^ (red and green circles, respectively, in Fig. 1h) were related to Si–O–Si asymetric stretching vibrations. The peaks at 970 and 800 cm^−1^ (blue and purple circles, respectively, in Fig. 1h) were described as a result of the Si–O in-plane and symmetrical stretching vibrations, respectively. These bands related to silica functionalization appeared also in the UCNPs-labeled aerogels thereby suggesting the correct incorporation of UCNPs into the 3D-printed aerogels.

**Fig. 2:**
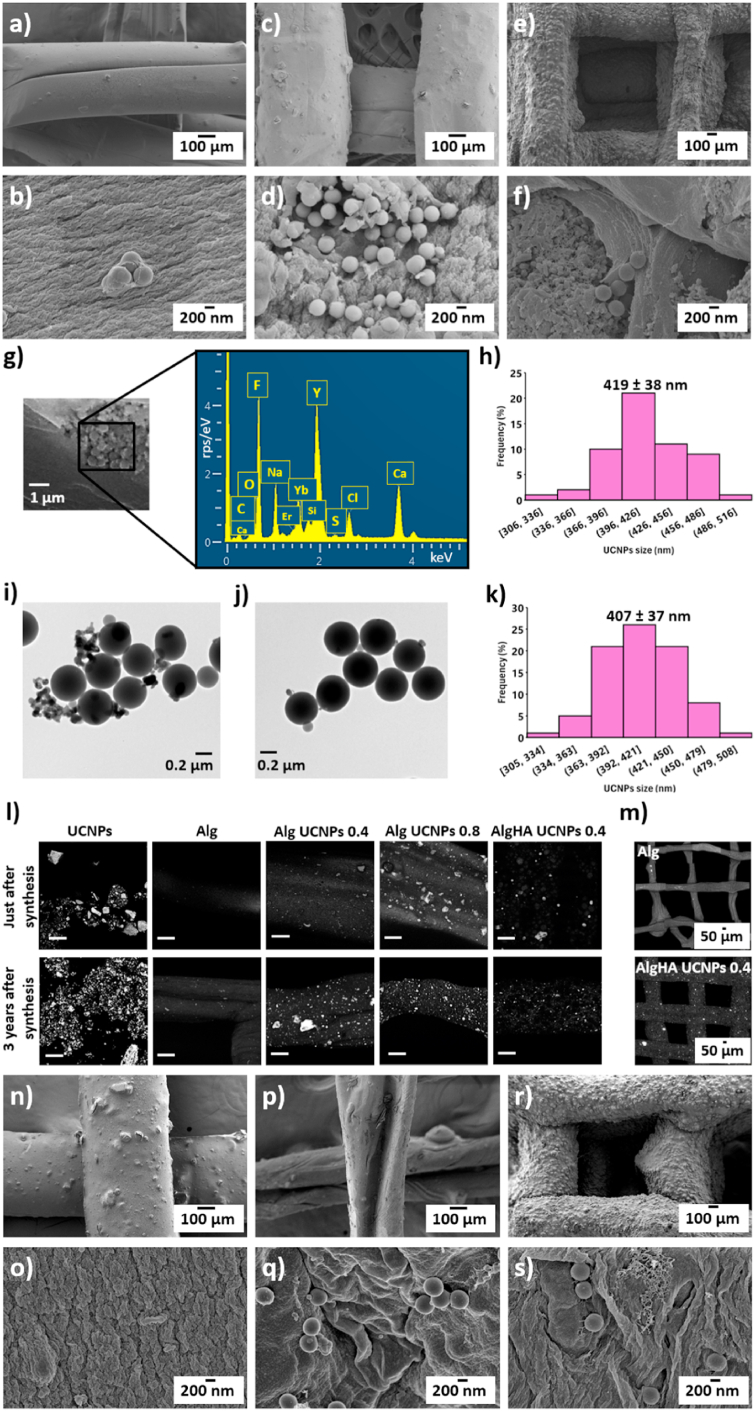
Microscopical analysis of UCNPs-decorated aerogels. SEM images of different aerogel formulations observed at two different magnifications: **(a,b)** Alg UCNPs 0.4; **(c,d)** Alg UCNPs 0.8; **(e,f)** AlgHA UCNPs 0.4. **(g)** EDX spectrum of Alg UCNPs 0.8 aerogels. **(h)** Particle size distribution of the UCNPs loaded in Alg UCNPs 0.8 aerogels. **(i, j)** TEM images of UCNPs. **(i)** UCNPs obtained just after synthesis; **(j)** UCNPs obtained 3 years after synthesis. **(k)** Particle size distribution of UCNPs. **(l,m)** Confocal images of different formulations of UCNPs-decorated aerogels (scale bar: 100 μm). **(n**–**s)** SEM images of different aerogel formulations 3 years after synthesis observed at two different magnifications: **(n,o)** Alg; **(p,q)** Alg UCNPs 0.8; **(r,s)** AlgHA UCNPs 0.4.

A homogeneous 3D-printed structure with strands aligned to form the designed grid pattern was observed by SEM imaging for all aerogel formulations (Fig. 2a,c,e). A highly porous structure with an interconnected fibrous network was observed for the different formulations studied (Fig. 2b,d,f). A similar smooth and open porous network was previously described for other 3D-printed alginate aerogels [18,36] and for hybrid alginate scaffolds aimed at tissue engineering applications [33,37]. A homogeneous distribution of UCNPs throughout the porous structure of aerogels was clearly observed. A relevant higher amount of UCNPs was visually detected for Alg UCNPs 0.8 with respect to the other formulations (Alg UCNPs 0.4 and AlgHA UCNPs 0.4) thus being consistent with the higher initial amount of UCNPs incorporated into the inks. Furthermore, the presence of UCNPs in the 3D-printed aerogels was confirmed by the EDX spectrum taken in a region of the aerogels with UCNPs (Fig. 2g). The peaks of the lanthanide ions initially used to synthesize the UCNPs (Y, Yb and Er) were clearly detected in the EDX spectra of the UCNPs-decorated aerogels, thus endorsing the correct doping of the aerogels with the UCNPs. Particle size distribution of the UCNPs just after synthesis displayed a normal distribution with a mean particle size of 419 ± 38 nm (Fig. 2h). A normal particle size distribution was also found after the analysis of the UCNPs incorporated into aerogels and located by SEM imaging, with a mean size of 407 ± 37 nm (Fig. 2k). The physicochemical integrity of the UCNPs was preserved after all the manufacturing process needed for the fabrication of the UCNPs-decorated aerogels.

Regarding textural parameters, the best values of A_BET_ were obtained when HA was not incorporated into aerogels regardless of the UCNPs content (Table 3). A decreasing tendency in A_BET_ and V_p_ were observed with increasing HA content, as previously described for AlgHA aerogels [18,19]. The doping of UCNPs in Alg aerogels slightly increased the A_BET_ at the lowest UCNPs concentration studied and this tendency was also preserved when HA was incorporated into aerogels. However, the A_BET_ values were maintained when working with the highest concentration of UCNPs.

**Table 3:** Textural properties of aerogels with or without UCNPs just after synthesis and after 3 years of storage. Notation: A_BET_, specific BET-surface area; d_p_, BJH-mean pore diameter; V_p_, BJH-specific pore volume; S, aerogels evaluated after 3 years of storage; ST, aerogels sterilized by the scCO_2_ method. * Data obtained from Ref. [18].

Formulation	A_BET_ (m^2^/g)	d_p_ (nm)	V_p_ (cm^3^/g)
Alg*	183 ± 9	19 ± 1	1.16 ± 0.06
AlgHA8*	118 ± 6	24 ± 1	0.99 ± 0.05
AlgHA16*	67 ± 3	26 ± 1	0.60 ± 0.03
AlgHA*	29 ± 2	31 ± 2	0.21 ± 0.01
Alg UCNPs 0.4	218 ± 11	19 ± 1	1.54 ± 0.08
Alg UCNPs 0.8	190 ± 10	18 ± 1	1.24 ± 0.06
AlgHA8 UCNPs 0.4	138 ± 7	24 ± 1	1.21 ± 0.06
AlgHA16 UCNPs 0.4	66 ± 3	28 ± 1	0.59 ± 0.03
AlgHA16 UCNPs 0.4 ST	48 ± 2	27 ± 1	0.38 ± 0.02
AlgHA UCNPs 0.4	49 ± 2	31 ± 2	0.50 ± 0.03
Alg S	80 ± 4	15 ± 1	0.40 ± 0.02
AlgHA8 S	67 ± 3	22 ± 1	0.46 ± 0.02
AlgHA16 S	53 ± 3	25 ± 1	0.41 ± 0.02
AlgHA S	39 ± 2	21 ± 1	0.23 ± 0.01
Alg UCNPs 0.4 S	123 ± 6	15 ± 1	0.65 ± 0.03
Alg UCNPs 0.8 S	82 ± 4	16 ± 1	0.45 ± 0.02
AlgHA8 UCNPs 0.4 S	76 ± 4	19 ± 1	0.49 ± 0.02
AlgHA16 UCNPs 0.4 S	39 ± 2	29 ± 1	0.34 ± 0.02
AlgHA UCNPs 0.4 S	29 ± 1	25 ± 1	0.18 ± 0.01

Stability studies of natural polymers are still scarce so the long-term stability (3 years) of 3D-printed aerogels was studied for the first time. After 3 years of storage, a decrease in A_BET_ and V_p_ was observed for Alg aerogels, but when HA was incorporated into the structures this effect was less marked. A decrease in textural properties of Alg aerogels was previously described after a storage of 3 months [38]. Stability studies of starch aerogels were performed with a marked decrease in A_BET_ and V_p_ observed after a shorter time (3 months) than the herein evaluated [39]. SEM images of aerogels 3 years after synthesis showed an external structure with the fibers still arranged to form the designed grid pattern (Fig. 2n,p,r). Also, the porous structure of the aerogels was mainly preserved, with the UCNPs homogeneously distributed through it (Fig. 2o,q,s). Stability of 3D-printed aerogels in terms of mid-term (3 and 7 months) maintenance of their textural performance were previously performed with good results for 3D-printed methylcellulose aerogels [16,17]. TEM images of UCNPs obtained just after synthesis exhibited a spherical shape and a homogeneous particle size distribution, comparable to that found after 3 years of storage at RT and protected from light (Fig. 2i and 2j, respectively). Confocal images of UCNPs-decorated aerogels (Fig. 2l and m) exhibited fluorescence with a similar intensity to the same formulations evaluated just after their synthesis [10], thus indicating the long-term maintenance of their fluorescence properties at least until 3 years after synthesis (Fig. S1). Entire aerogel scaffolds were imaged in the fluorescent mode to further confirm these results (Fig. S2). Data endorsed the physicochemical and functional stability of the theranostic implants after 3 years at the conditions of the study (storage at RT in closed plastic jars). Stability studies of the 3D-printed aerogels under harsher conditions than the herein reported could be performed as part of future work to unveil the textural properties dependence on the storage conditions.

The use of scCO_2_ as an adequate method for the sterilization of UCNPs-decorated aerogels was assessed by BET and confocal microscopy analyses. Similar results in terms of textural and fluorescence performance were obtained before and after scCO_2_ post-processing step (Table 3 and Fig. S3, respectively), thus endorsing the suitability of this method for the sterilization of UCNPs-decorated aerogels.

### 3.3 Cell studies

**Fig. 3:**
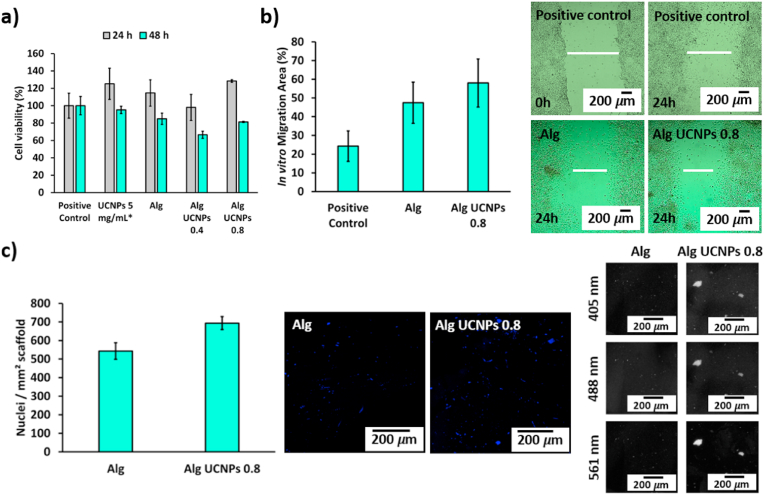
Cellular studies of different aerogel formulations. **(a)** Cell viability test on BALB/c 3T3 cells. Viability (expressed in %) of BALB/c 3T3 cells after 24 and 48 h in contact with aerogels measured by WST-1 test. Positive controls: BALB/c 3T3 cells without contact with UCNPs or aerogels. * Data obtained from Ref. [10]. **(b)** Scratch test on BALB/c 3T3 cells. Quantitative analysis (expressed in %) of the BALB/c 3T3 cells migration area after 24 h in cell culture and microscopy images after 0 and 24 h. Positive controls: BALB/c 3T3 cells without contact with UCNPs or aerogels. **(c)** Cell attachment test on MSCs. Quantitative analysis (expressed in nuclei number per mm^2^ of scaffold) after 13 days in cell culture (left) and confocal images of DAPI-stained MSCs seeded on aerogels (middle) and of aerogels after cell attachment tests (right). No statistically significant differences among groups were obtained after post hoc Tukey HSD multiple comparison test (*p* < 0.05).

UCNPs-labeled aerogels are bifunctional structures able to maintain the biocompatibility of the implant. High values of cell viability were observed after 24 and 48 h of contact with different aerogel formulations (Fig. 3a). No statistically significant differences were found between the cell viability values of positive controls and fibroblasts after contact with UCNPs-loaded aerogels, indicating that implants did not induce any cytotoxic effects on BALB/c 3T3 cells, thereby being biocompatible [40]. These are good results since neither the implant itself nor the UCNPs, that could be released into the culture medium after 24 or 48 h, affect the BALB/c 3T3 cells’ viability. In contrast, certain metals incorporated into implants without being part of a nanocomplex were reported to neglect the biocompatibility of the end structure [7]. Other core-shell UCNPs also protected with a silica coating were cytotoxic after 24 h above a certain concentration (100 μg/mL) [41]. The herein synthesized UCNPs were *in vitro* tested beyond this threshold (until 5 mg/mL) with good results in terms of biocompatibility, thereby suggesting their safety use [10].

Cell migration capability into the wound site during the inflammatory phase of defect repair is essential for tissue repair [15]. Similar values of migration area were measured for fibroblasts after contact with aerogels labeled or not with UCNPs, without statistically significant differences between both groups (Fig. 3b). Moreover, no relevant differences were observed between the MSCs adhesion to a control scaffold (Alg) and to an UCNPs-labeled one (Alg UCNPs 0.8) (Fig. 3c). It has been reported that UCNPs can significantly influence long-term cell functionality [42], but the herein obtained results suggest the lack of impact of the synthesized UCNPs on cell migration or adhesion processes. Confocal images of UCNPs-decorated aerogels exhibited fluorescence at the characteristic wavelengths of the upconversion process undergone by UCNPs (405, 488 and 561 nm), showing that nanocomplexes are still present and functional in the theranostic system after the test (13 days). These are good results since it was previously reported that UCNPs in aqueous environments could not be detected in the long-term due to the intensity loss of the upconversion process after only several hours were elapsed [43].

### 3.4 Hemocompatibility studies

**Fig. 4:**
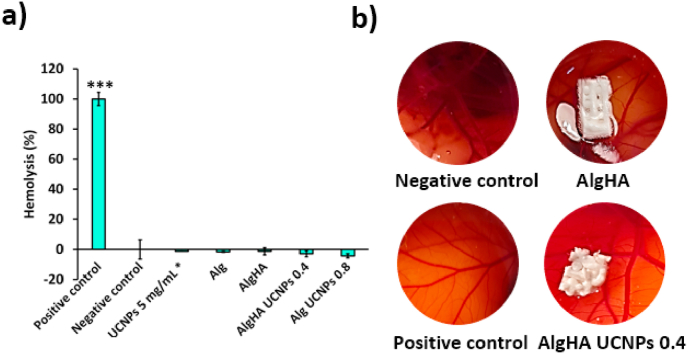
Hemocompatibility evaluation of different aerogel formulations. **(a)** Hemolysis percentage of UCNPs and UCNPs-labeled aerogels. Positive control: 4 % v/v Triton X-100 and negative control 0.9 % w/v NaCl. * Data obtained from Ref. [10]. Statistically significant differences among groups were denoted as *** (post hoc Tukey HSD multiple comparison test, *p* < 0.001). **(b)** HET-CAM test of aerogels. Negative control: 0.9 % w/v NaCl, positive control: 0.1 N NaOH.

Interactions between blood and the implant after the initial trauma could lead to a localized edema at the implantation site [44,45]. No hemolytic activity was detected upon contact of the different aerogel formulations with human blood (Fig. 4a). Results endorse the properties of these aerogels as scaffolds sought to minimize the foreign body reaction [46]. Moreover, *in ovo* HET-CAM assays are a suitable method to predict tissue toxicity [47]. No hemorrhage, clotting, vascular lysis, or vessel bleeding were observed when UCNPs-labeled aerogels contacted the CAM (Fig. 4b) for the standard test time (5 min), with similar results to those observed for the positive control. Similar results were observed for polycaprolactone (PCL) and for methylcellulose-based scaffolds [16,17,47]. Both tests suggest the hemocompatibility of the formulations with biological tissues.

### 3.5 *In vivo* safety studies

#### 3.5.1 Determination of plasmatic cytokines

**Fig. 5:**
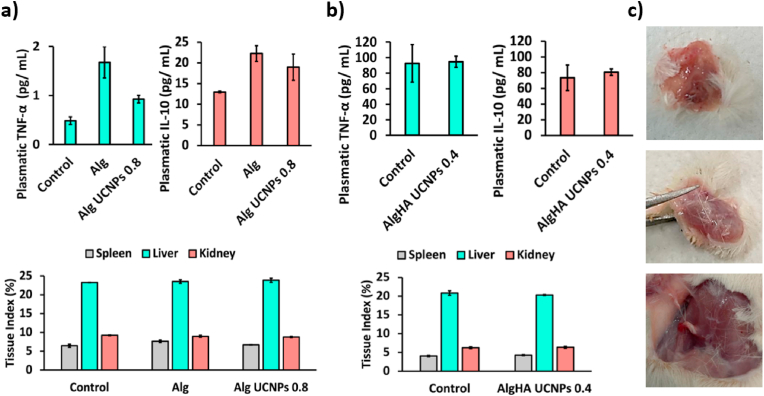
Plasmatic cytokines (TNF-α and IL-10) concentration (expressed in pg/mL) and tissue index (of spleen, liver, and kidney) values (expressed in %) of **(a)** mice and **(b)** rats 1 week after subcutaneous implantation of different aerogel formulations (Alg, Alg UCNPs 0.8 or AlgHA UCNPs 0.4, respectively). Control: animals with a surgical incision without scaffold implantation. Plasmatic concentration of IL-6 was not detected. No statistically significant differences among groups were obtained after post hoc Tukey HSD multiple comparison test (*p* < 0.01). **(c)** Images of the connective tissue capsule developed 1 week after subcutaneous implantation of UCNPs-labeled aerogels (AlgHA UCNPs 0.4).

Polymeric devices commonly induce inflammation after implantation and an immunologic response through a foreign body reaction [48]. Immune cells express pro-inflammatory cytokines such as TNF-α, IL-1 and IL-6 or anti-inflammatory cytokines like IL-10 and transforming growth factor β [49]. These biomarkers could differentiate the physiological healing from the pathological fibrosis [50,51]. *In vitro* downregulation of IL-6 and TNF-α was described as an anti-inflammatory response [52]. In fact, inflammation is mimicked *in vitro* by incorporating these cytokines in a very low concentration (ng/mL levels) to cell culture medium [15]. For these reasons, the expression of IL-6 and TNF-α is commonly studied after scaffold implantation [53]. An anti-inflammatory microenvironment characterized by low expression of pro-inflammatory cytokines was observed after 1-week subcutaneous scaffold implantation in mice and rats. In this work, all groups presented similar levels of TNF-α and IL-10 (Fig. 5a and b) and no detectable IL-6 levels were obtained. A low level of pro-inflammatory and anti-inflammatory cytokines reported on the tissue surrounding a bone implant was related to the inflammation regression and the long-term tissue growth [49,53].

Potential toxicity of aerogels was also evaluated by the tissue index values without observing statistically significant differences among the different groups studied (Fig. 5a and b), thus suggesting the lack of organ toxicity [8]. All these results are aligned with the detected low production of cytokines. IL-6 and TNF-α are up-regulated after implantation, while IL-10 is up-regulated in the later stage of the foreign body reaction [44].

All implant sites exhibited a complete wound healing after the different *in vivo* studies. No adverse effects appeared in terms of infection or inflammation signs around the surgical area, with sutures still present in all animals. Ciprofloxacin is commonly used to prevent infection after implantation of scaffolds [54], but antibiotics were not needed in this study. For bioimaging assay, where staples were used instead of sutures, wound was completely closed without a relevant scar. Similar results in terms of visual inspection were found after a similar surgical intervention [53]. The final part of the foreign body reaction is the fibrous capsule formation around the implant that ends with the isolation of the biomaterial from the local environment [44]. A connective tissue capsule was observed immediately around the implant in all cases (with or without UCNPs) (Fig. 5c) suggesting that the presence of UCNPs did not interfere with the normal wound healing process. Finally, all implants were visually inspected by removal after sacrifice. Structure biodegradation was more significant after the bioimaging assay than after safety test due to the longer duration of the experiment (1 week vs 3 weeks).

#### 3.5.2 Histological evaluation of major organs

**Fig. 6:**
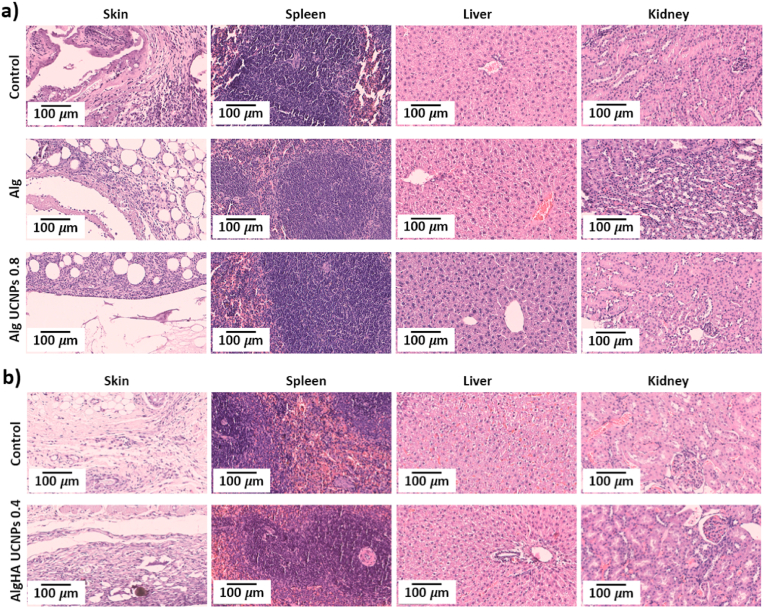
Histological images obtained by H&E staining of different tissues (skin, spleen, liver, and kidney) of **(a)** mice and **(b)** rats 1 week after subcutaneous implantation of different aerogel formulations (Alg, Alg UCNPs 0.8 or AlgHA UCNPs 0.4, respectively). Control: animals with a surgical incision without scaffold implantation.

All animals presented a subcutaneous granulomatous inflammatory reaction surrounding the implant, composed of inflammatory infiltrates rich in macrophages, lymphocytes and plasma cells (Fig. 6a and b). Control animals (without scaffold implantation) exhibited a minimal scar reaction, with epidermal hyperplasia and a minimal inflammatory reaction secondary to the incisional surgical procedure. As control animals also showed tissue damage, the observed foreign body reaction could be a response to the silk suture rather than to the presence of the implant [55]. The inflammatory response is an expected secondary reaction to the presence of a foreign body and could be part of the healing process and play an important role in other physiological processes like osteogenesis [56]. It should be noted that the HA-loaded implants herein tested have previously demonstrated to be long-term bioactive and promoters of cell adhesion and migration, so they were proposed as bone tissue engineering scaffolds [18,19]. Implants coatings or strategies to trigger macrophage polarization have been developed to promote an adequate foreign body reaction, but a similar response was observed in the skin surrounding PCL scaffolds with cellular exosomes incorporated to minimize the foreign body reaction [46,56].

Regarding the other organs studied, no morphological abnormalities were identified, and they were histologically similar to the controls. Similar results in terms of main organ histology were reported after subcutaneous implantation of 3D-printed PCL-based scaffolds while necrosis clearly appeared in the H&E histological sections of the same tissues when photothermal therapy was applied [57]. Isolated UCNPs or UCNPs aggregates were not histologically detected on liver, kidney, and spleen thus suggesting that they were not accumulated on these organs. The same organs were histologically examined after subcutaneous implantation of PCL scaffolds loaded with gold nanoclusters in mice for 4 week without relevant differences with respect to the controls [8]. Aggregates were also searched in the same major organs without detecting them, so it was hypothesized that nanocomplexes did not form aggregates *in vivo* and could be eliminated by urine.

A similar *in vivo* biocompatibility study was reported for silica aerogels without observing remarkable changes after histopathological evaluation of the major organs [58]. On the nearby tissue, a mild inflammatory response and fibrosis were also detected until twenty months after surgery, which was described as part of the natural foreign body reaction after implantation of foreign materials. Overall, results indicate the good biocompatibility of the UCNPs-labeled aerogels 1 week after subcutaneous implantation in mice and rat and suggest that the granulomatous reaction is a local response. An absence of infection signs was observed in all animals of the *in vivo* studies, thus suggesting the effectiveness of the scCO_2_ with the additive H_2_O_2_ as a sterilization method for implantable devices.

### 3.6 *In vivo* bioimaging studies

**Fig. 7:**
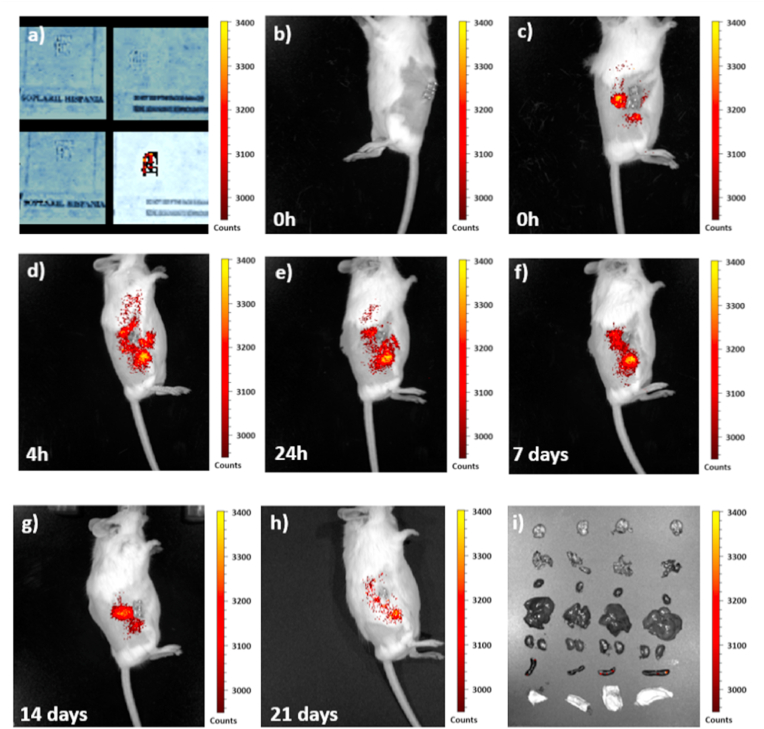
Fluorescence intensity maps (expressed in normalized counts) obtained at the excitation wavelength of 745 nm and the emission wavelength of 840 nm. **(a)** Alg (left) and Alg UCNPs 0.8 aerogel implants (right) without (top) and with (bottom) fluorescence excitation. **(b)***in vivo* fluorescence image of Alg implant (control of fluorescence emission). **(c**–**h)***in vivo* fluorescence images recorded at different timepoints post-implantation of mice bearing Alg UCNPs 0.8 aerogel implants. **(i)** Fluorescence image of excised brain, liver, heart, lungs, kidneys, spleen, and skin surrounding the implant (rows from top to bottom) of a control animal (without scaffold implantation) and of the 3 animals with Alg UCNPs 0.8 theranostic implant (columns from left to right).

Fluorescence intensity was homogeneously distributed throughout the UCNPs-labeled aerogels (Fig. 7a). In contrast, no fluorescence was detected in the non-loaded aerogels before (Fig. 7a) or after (Fig. 7b) *in vivo* subcutaneous implantation by the setup used for the bioimaging assay. Fluorescence was specifically accumulated only in the area where the theranostic nanodevice was implanted for all the assay duration (Fig. 7c–h). The ability to *in situ* monitor aerogels once implanted was possible from the moment of implantation until 21 days after the surgery in a real-time and non-invasive manner. Fluorescence trend showed an increase in the fluorescence intensity 4 h after implantation, probably due to a high initial release of UCNPs in response to the start of the biodegradation of the aerogel scaffold (Fig. S4). Then, the fluorescence intensity increased over time and a lower fluorescence accumulation in the implant site was detected only in the last timepoint (21 days). This decrease in the fluorescence recorded was probably due to the progress of the biodegradation of the implant that could leach UCNPs from the aerogel thereby decreasing the fluorescence intensity and monitoring capability from this moment on. The tissue engineering process undergone by the UCNPs-labeled scaffold starts just after implantation and proceed until the complete resorption of the structure. It should be noted that after the duration of the bioimaging assay (21 days) aerogels had undergone a partial resorption but were still detectable by fluorescence-based bioimaging. If the lesion sought to be regenerated by the scaffold needs more time to be completely recovered, further adjustments at biomaterial and 3D-printing levels are required to warrant longer biodegradation times before the theranostic device can be tested in a clinical setting.

Only the spleen of animals implanted with UCNPs-decorated aerogels displayed a very low fluorescence intensity, probably due to the low concentration of UCNPs initially incorporated into aerogels and to the clearance of part of these UCNPs during the 21 days of the study. The fluorescence intensity detected at spleen level could indicate the involvement of this tissue in the metabolism of UCNPs or the accumulation of the nanocomplexes at that level. Further experiments at different timepoints must be performed to unveil the biodistribution profile of the UCNPs once the theranostic device is implanted.

In other study [8], the *in vivo* degradation of poly-lactic-*co*-glycolic acid (PLGA) scaffolds was traced by detecting a relevant decrease in the recorded fluorescence intensity 4 weeks after implantation. Fluorescence was recorded for longer time in that case than in the herein reported work, probably due to the slower *in vivo* biodegradation rate of the polymer forming the scaffold (PLGA vs alginate). Furthermore, the long-term high fluorescence of the UCNPs could enhance their monitoring capability, thereby increasing the sensitivity of the bioimaging approach. Consistently, due to the high sensitivity of the upconversion process undergone by UCNPs, these nanocomplexes have been employed for *in vivo* cell tracking by fluorescence bioimaging [59]. Alkaline phosphatase (ALP) was targeted by a molecular probe loaded in PCL scaffolds to thus induce fluorescence and turn the implant monitorable for 4 weeks [60]. However, ALP is not an exclusive biomarker of the bone tissue and was previously related to different pathological pathways. Moreover, cytotoxicity of the molecular probe must be confirmed for their safety use [61].

Ultrasounds technique was previously employed for the *in vitro* and *ex vivo* degradation of polyurea-crosslinked alginate and silica aerogels [62,63]. An *in vivo* strategy was also described, but the animals were sacrificed before the implantation surgery, so the *in vivo* traceability over time was not studied in that case [64]. It should also be noted that the phosphor compound that enable the tracing must be doped at high contents (15 and 50 wt% of phosphor with respect to the aerogel) to render traceable aerogels [65,66]. A strong dependance with the dopant concentration was described in that case in contrast to the herein synthesized UCNPs, thereby suggesting the UCNPs-decorated aerogels developed in this work as a safer alternative for the *in vivo* long-term monitoring of implants.

## 4 Conclusions

Bifunctional implants were successfully manufactured by the doping of 3D-printed scaffolds with highly fluorescent UCNPs. Nanodevices obtained were *in vivo* traceable until 3 weeks after implantation thus enabling, for the first time, the *in vivo* monitoring of aerogels in a safe and non-invasive way. UCNPs present stable photophysical properties that are preserved after their incorporation into aerogels and suitable for bioimaging. A homogeneous distribution of UCNPs was observed throughout the pores of the 3D-printed aerogels. UCNPs-decorated aerogels are biocompatible structures able to promote cell adhesion and cell migration. Results indicate a minimal systemic toxicity without relevant inflammatory response in terms of cytokine expression, tissue index and histology of main organs after subcutaneous implantation of the theranostic device. Specific metabolic pathways involving these UCNPs-labeled aerogels could be studied in the future by *in vivo* bioimaging experiments in a time- and dose-dependent manner to determine their internalization and excretion process. This work opens up the possibility of studying *in vivo* the biodegradation, resorption rates and toxicity of UCNPs-decorated aerogels. Properties of the scaffolds after long-term implantations can also be studied. Tissue regeneration process could be thereby *in situ* monitored as well as the scaffold position and state providing useful information for disease treatment in real-time by *in vivo* fluorescence imaging.

## Ethics approval and consent to participate

Wistar rats (male, 200–300 g) from Charles River (Barcelona) and mice BALB/c (male, 20–30 g) from IGC (Lisbon) were housed and maintained in compliance with the guidelines outlined in the Guide for the Care and Use of Laboratory Animals, in accordance with the national (DL 113/2013, 2880/2015, 260/2016 and 1/2019) and international (Directive 2010/63/EU) accepted principles for laboratory animals' use (3 R's principles). Safety studies were reviewed and approved by the DGAV (national authority) and by Animal Experiment Ethics Committee (ORBEA) of the Faculty of Pharmacy of University of Lisboa following the Declaration of Helsinki.

## Declaration of interest

All authors disclose no actual or potential conflict of interest related with any financial and personal relationships with other people or organizations that could inappropriately influence, or be perceived to influence, this work.

## Funding

This work was funded by MICIU/AEI/10.13039/501100011033 [Grants PID2020-120010RB-I00 and PDC2022-133526-I00] and by ERDF/EU and European Union NextGenerationEU/PRTR. This work was also supported by Fundação para a Ciência e Tecnologia (FCT), doi:10.54499/UIDB/00645/2020, doi:10.54499/UIDP/00645/2020, UIDB/04138/2020, UIDP/04138/2020, doi:10.54499/UIDB/00102/2020 and doi:10.54499/UIDP/00102/2020. The work was carried out in the framework of the COST Innovators Grant IG18125 “Technical, commercial and societal innovations on aerogels towards circular economy” (ECO-AERoGELS) funded by the European Commission. A.I.-M. acknowledges the AERoGELS COST Action (ref. CA18125) for the granted Short Term Scientific Mission to synthesize the UCNPs in the Universidade de Coimbra and to the Xunta de Galicia for her predoctoral research fellowship [ED481A-2020/104]. Authors acknowledge Doctor José Rino for the microscopic facilities at iMM - Instituto de Medicina Molecular, Lisbon and Carmen Álvarez-Lorenzo for the rheological studies of the inks at University of Santiago de Compostela.

## Data availability

Research data are available upon request to the authors.

## CRediT authorship contribution statement

Ana Iglesias -Mejuto: Writing – original draft, Writing – review & editing, Methodology, Investigation, Formal analysis, Data curation, Conceptualization.

Rui Pinto: in vivo Methodology.

Pedro Faísca: in vivo Methodology.

José Catarino: in vivo Methodology.

João Rocha: in vivo Methodology.

Luisa Durães: Writing – review & editing, Supervision , Resources, Project administration, Funding acquisition, Conceptualization.

Maria Manuela Gaspar: Resources, in vivo Methodology, Funding acquisition.

Catarina Pinto Reis: Resources, in vivo Methodology, Funding acquisition.

Carlos A. García -González: Writing – review & editing, Supervision, Resources, Project administration, Methodology, Funding acquisition, Conceptualization.
