# Supplementary material for: 3D-printed aerogels as theranostic implants monitored by fluorescence bioimaging
Source: Bioact Mater. 2024 Aug 8;41:471–84. doi: 10.1016/j.bioactmat.2024.07.033 (PMC11364008; doi:10.1016/j.bioactmat.2024.07.033)
Supplement: Multimedia component 1 [file mmc1.docx]

**Supplementary Information**

**3D-printed aerogels as theranostic implants monitored by fluorescence bioimaging**


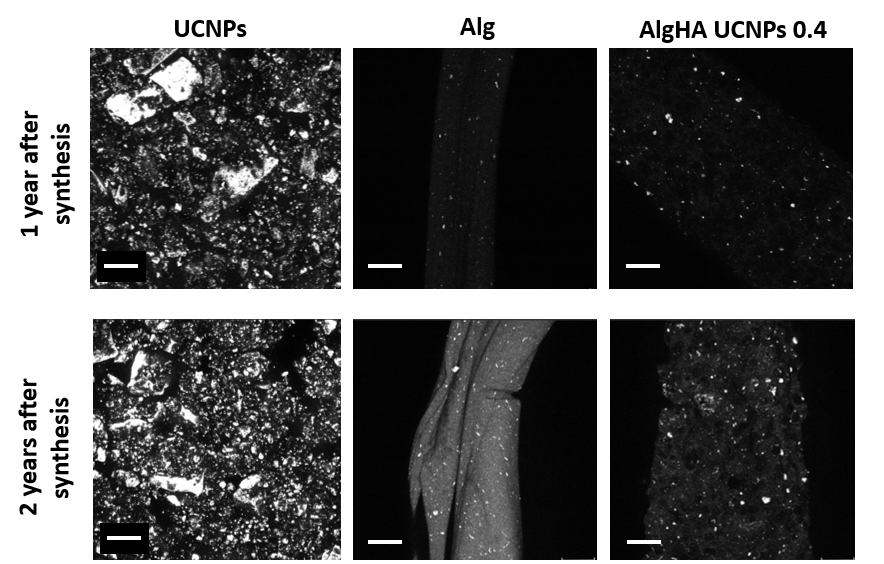


**Figure S1:** Confocal images of UCNPs, Alg and AlgHA UCNPs 0.4 aerogels obtained 1 and 2 years after their synthesis (scale bar: 100 µm).


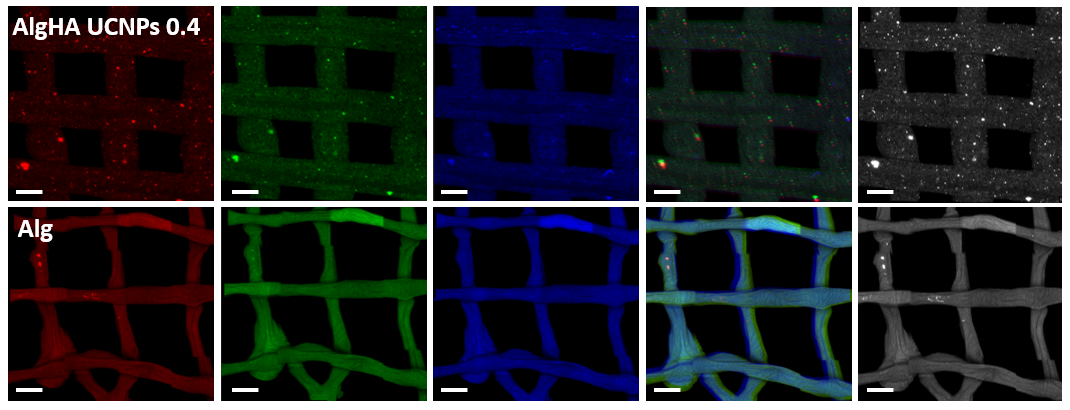


**Figure S2:** Confocal images in fluorescent mode of Alg and AlgHA UCNPs 0.4 aerogels. Images were collected at different wavelengths: 405 nm (red), 488 nm (green) and 561 nm (blue) (scale bar: 0.5 mm).


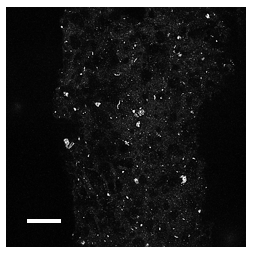


**Figure S3:** Confocal image of Alg HA16 UCNPs 0.4 aerogels after the sterilization post-processing step with scCO_2_ (scale bar: 100 µm).

**
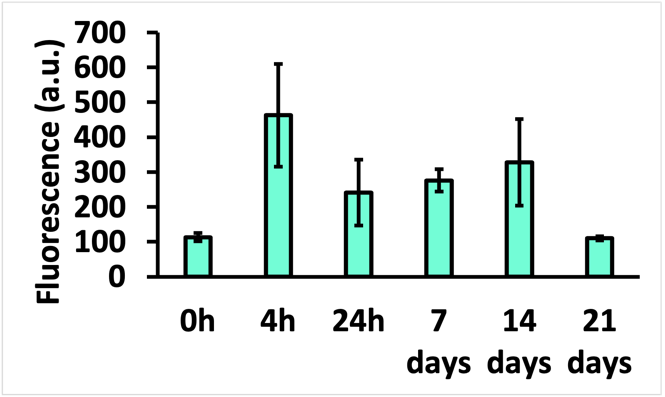
**

**Figure S4:** Fluorescence intensity profile of *in vivo* bioimaging study (21 days).
